# Safety of laparoscopic oophorectomy in a Dutch national pediatric cancer cohort

**DOI:** 10.1016/j.xfre.2025.11.008

**Published:** 2025-12-02

**Authors:** Nikita H.Z. Clasen, M.E. Madeleine van der Perk, Marianne D. van de Wetering, Annelies M.E. Bos, Anne-Lotte L.F. van der Kooi, Kim van Loon, Margreet A. Veening, Irene M. IJgosse, Sebastian J.C.M.M. Neggers, Martine van Grotel, Dorine Bresters, Hanneke M. van Santen, Willem M.J.A. Verpoest, Leendert H.J. Looijenga, Brigitte Arends, Jeanette van Leeuwen, Simone L. Broer, Alida F.W. van der Steeg, Marry M. van den Heuvel-Eibrink

**Affiliations:** a Princess Máxima Center for Pediatric Oncology, Utrecht, the Netherlands; b Department of Obstetrics and Gynecology, Leiden University Medical Center, Leiden, the Netherlands; c Department of Anesthesiology, University Medical Center Utrecht, Utrecht, the Netherlands; d Department of Internal Medicine, Section Endocrinology, University Medical Center Rotterdam Erasmus MC, Rotterdam, the Netherlands; e Department of Pediatric Endocrinology, University Medical Center Utrecht - Wilhelmina Children's Hospital, Utrecht, the Netherlands; f Department of Reproductive Medicine and Gynecology, University Medical Center Utrecht, Utrecht, the Netherlands; g Department of Pathology, University Medical Center Utrecht, Utrecht, the Netherlands; h Division of Child Health, University Medical Center Utrecht - Wilhelmina Children’s Hospital, Utrecht, the Netherlands

**Keywords:** Childhood cancer, fertility preservation, oophorectomy, safety

## Abstract

**Objective:**

To report our experiences on the safety of laparoscopic oophorectomy (LO) for ovarian tissue cryopreservation in childhood cancer (CC) patients.

**Design:**

Descriptive study of a prospectively registered cohort.

**Subjects:**

All CC patients undergoing LO in the Netherlands from November 2020 until April 2024.

**Exposure:**

Laparoscopic oophorectomy for means of fertility preservation before or during gonadotoxic treatment.

**Main Outcome Measures:**

Safety, including procedure-related complications, such as critical events (desaturation, bradycardia), need for mechanical ventilation in a pediatric intensive care unit (PICU), site infections, hemorrhage, and survival, scored according to Clavien Dindo (CD) classification.

**Results:**

Eighty-eight patients, median age 6.5 years (range 1–18 years) underwent LO, of which 2/88 patients developed CD grade 4, 1/88 CD grade 3, and 8/88 CD grade 2 complications, with the most common being port site infection (6%) and bleeding (6%). No clear determinant could be identified as a risk factor for complications. All patients recovered from procedure-related complications and no patient died as a consequence of oophorectomy.

**Conclusion:**

Laparoscopic oophorectomy is a relatively safe procedure to preserve future fertility for girls with cancer with a high risk of gonadal failure and consequent infertility. However, LO can be accompanied by complications in children with cancer. Therefore, safety monitoring and identification of risk factors for complications based on larger cohorts in future studies are needed. Prevention of complications of LO is an important obligation to balance the future benefits against the risks of LO to enhance the future chance of pregnancy for girls with cancer.

## Introduction

Over the past decades, survival rates of children with cancer have increased to over 80% (1, 2, 3). This has raised awareness for short and long-term toxicity after childhood cancer treatment. Gonadal damage and subsequent infertility has been reported to be one of the most relevant late effects for patients, parents, childhood cancer survivors, and health care experts (4), as this seriously impairs quality of life (5, 6). High quality oncofertility care is therefore an important mission in current pediatric oncology practice, which attempts to enable future fertility, reproductive health, and to preserve the best quality of life for the future in girls with cancer with a high risk of gonadotoxicity (7, 8). In adult settings, fertility counseling, gonadal tissue cryopreservation and subsequent auto-transplantation have led to over 200 live births in (young) adult women with cancer (Supplemental Table 1, available online). The American Society of Reproductive Medicine (ASRM) stated in 2019 that ovarian tissue cryopreservation (OTC) should be offered as standard of care in all children and young adolescents with cancer with a high gonadal damage risk (9, 10), which has been an important step in the international development of allowing OTC in prepubertal girls in need for (immediate) gonadotoxic treatment. In children with cancer, results of live births after 46 auto-transplantations of cryopreserved ovarian tissue, harvested before the age of 19 years for restoration of fertility, are reported (11, 12). Out of 46 auto-transplantations, 20 pregnancies were achieved, of which 16 resulted in successful live births of healthy born children (11, 12), 2 after auto-transplantation of prepubertal (11) and 14 after auto-transplantation of postpubertal harvested ovarian tissues (11, 12). This contributes to the fact that enthusiasm toward laparoscopic oophorectomy (LO) for fertility preservation in girls with cancer has started to overrule past skepticisms. However, at the moment, only limited well-documented information is available on the safety of the oophorectomy procedures in this relatively *novel* setting of young girls with cancer. Although available reports in (young) adults (13, 14, 15, 16, 17, 18, 19, 20, 21) showed reassuring results, published evidence on safety of LO in children with cancer (22, 23, 24, 25, 26, 27, 28, 29, 30, 31, 32, 33, 34) is mostly retrospective, scarce, and not based on national cohorts.

Therefore, the current study aimed to evaluate and describe the safety of LO procedures in a carefully stratified, triaged and counseled prospective cohort of girls with cancer in the first years of our national centralized pediatric oncology center in the Netherlands, as part of the prospective PEARL ("PresErving ovARian function through cryopreservation and informing girLs with cancer about infertility due to gonadotoxic treatment") study. We aimed to evaluate procedure-related complications to improve the safety of the procedure, to be able to weigh the future benefits against the risks of LO for individual girls with cancer.

## Materials and methods

### Patients

Between November 2020 and April 2024, all girls (1–18 years) newly diagnosed with pediatric cancer or a relapse of pediatric cancer, treated at the Princess Máxima Center, a national center for pediatric oncology, were identified, triaged, and informed regarding their risk of gonadal damage, based on a national 5-step oncofertility care approach (8). For this purpose, a personalized gonadal damage risk was estimated using the anticipated treatment-based gonadal risk stratification tool (8), in line with current international guidelines (5, 8, 35, 36, 37). Following this approach, girls with a high risk of gonadal damage were offered counseling by a dedicated gynecologist with fertility expertise in childhood cancer patients. In each case OTC was chosen as a fertility preservation option by shared decision making; complication-related data were prospectively registered and analyzed for the current descriptive study.

In our center, as per LO, 2 surgical experts (in principle 1 surgeon, 1 gynecologist) together perform the procedure. From both our surgical-oncological and gynecological teams, only 3 surgeons and 3 gynecologists specialized in oncofertility perform these procedures in our center, to guarantee both surgical-oncological and gynecological expertise. At present, no international consensus on the surgical technique for OTC exists (11). Nonetheless, unilateral complete oophorectomy is most frequently applied across centers worldwide, and this was the surgical technique implemented in the current study. In the indicated time period, in our national pediatric oncology center, LO was not recommended for children below the age of 13 months (38, 39) as of yet, following the national anesthetic guideline for elective laparoscopic procedures for infants (40).

### Methods

On informed consent, registered data included patient demographics, tumor type, (timing of) treatment, and details on the individual oophorectomy procedures, including whether the procedure was combined with other procedures under anesthesia or as a stand-alone procedure. To prevent delaying the start of oncological treatment, oocyte harvest was not pursued in any of the postpubertal patients in this cohort. Patient’s condition before surgery was registered, based on preceding chemotherapy, preoperative blood counts including transfusions, the use of preoperative prophylactic antibiotics (41), and registered vital functions in the 24 hours preceding LO by means of the pediatric early warning signs (PEWS) (42) (Supplemental Table 2).

For scoring of oophorectomy-related complications, the Clavien Dindo (CD) classification (Table 1) was used (43), an internationally standardized surgical complication classification that is easy and effective to use in daily practice in children. Oophorectomy-related complications included all complications that could not be related to another (surgical) procedure, including critical events, defined as CD grade ≥3 cardiorespiratory or neurological events (such as desaturation, bradycardia, and seizures), and possible need for mechanical ventilation in a pediatric intensive care unit (PICU). Also, data regarding port site infections (i.e., port of the laparoscopic trocar), hemorrhage (including intraabdominal bleeding, port site bleeding or hematoma), and ultimate survival data at the last moment of follow-up were collected. The timing of related complications was registered. Complications on the day of oophorectomy were stratified in "day 0 during procedure," which included all pre- and intraoperative complications, and "day 0 after procedure," which included all postoperative complications after extubation in the first 24 hours after the procedure. For postoophorectomy complications, the day of the start of chemotherapy after oophorectomy procedure was documented as a possible determinant for the occurrence of complications after oophorectomy. Oophorectomy-related complications were registered until 30 days after the procedure.

**Table 1:** Clavien Dindo classification of surgical complications (43).

Grade	Definition
Grade 1	Any deviation from the normal postoperative course without the need for pharmacological treatment or surgical, endoscopic, and radiological interventions Allowed therapeutic regimens are drugs as antiemetics, antipyretics, analgetics, diuretics, electrolytes, and physiotherapy. This grade also includes wound infections opened at the bedside
Grade 2	Requiring pharmacological treatment with drugs other than such allowed for grade I complications Blood transfusions and total parenteral nutrition are also included
Grade 3	Requiring surgical, endoscopic or radiological intervention
Grade 3a	Intervention not under general anesthesia
Grade 3b	Intervention under general anesthesia
Grade 4	Life-threatening complication (including CNS complications^a^) requiring IC/ICU management
Grade 4a	Single organ dysfunction (including dialysis)
Grade 4b	Multiorgan dysfunction
Grade 5	Death of a patient

CNS = central nervous system; IC = intermediate care; ICU = intensive care unit.

a Including brain hemorrhage, ischemic stroke, subarachnoid bleeding, but excluding transient ischemic attacks.

### Ethical approval

Ethical approval was obtained from the METC Utrecht (Medical Ethical Committee Utrecht) for the PEARL ("PresErving ovARian function through cryopreservation and informing girLs with cancer about infertility due to gonadotoxic treatment") study (METC research file NL72115.041.19, METC-protocol number 19/783, Netherlands trial register number NL8192).

## Results

### Patient demographics

From November 2020 until April 2024, 88 girls, with a median age at diagnosis of 6.5 years (range 0–18 years), underwent LO (Table 2), 75 patients (85%) with a primary malignancy, and 13 (15%) at the time of a relapse. Eighteen patients (20%) underwent LO before the start of cancer treatment, the other patients underwent oophorectomy between chemotherapy courses. Ovarian tissue cryopreservation had been offered based on expected cyclophosphamide equivalent dose (CED)-score (CED ≥6 g/m^2^) (36) (n = 53, 60%), approaching conditioning and/or total body irradiation (TBI) and hematopoietic stem cell transplantation (HSCT) (n = 19, 22%), necessity of whole abdominal radiotherapy (n = 1, 1%), or combined indications (n = 15, 17%) (Table 2). For patients with prior chemotherapy exposure, the median time since the last day of chemotherapy course preceding oophorectomy was 14 days. Thirty-eight procedures (43%) had been combined with other interventions, such as central line insertions (n = 28, 32%), bone marrow biopsy (n = 13, 15%), bronchoalveolar lavage (BAL) (n = 12, 14%), and lumbar punctures (n = 6, 7%) (Table 2).

**Table 2:** Characteristics of patients with and without laparoscopic oophorectomy-related complications (November 2020–April 2024).

Characteristic	All patients (n = 88)	Patients without complications (n = 77)	Patients' related complications (n = 11)					
			CD Grade 2			CD Grade 3–4		
			Port site bleeding (n = 5)	Port site infection (n = 4)	Desaturation (n = 1)	Bradycardia (n = 1)	Failure to wean (n = 1)	Port site abscess (n = 1)
Median age at time of diagnosis in years (range)	6.5 (0–18)	7.0 (0–18)	2.0 (2–9)	9.5 (6–14)	1.0	6.0	2.0	9.0
Disease characteristics								
Hemato-oncology (%)	**26** (**30)**	**21** (**27)**	**1** (**20)**	**3** (**75)**	**–**	**1** (**100)**	**–**	**–**
ALL					**–**		**–**	**–**
Primary	6 (7)	5 (6)	**–**	1 (25)		**–**		
Relapse	4 (5)	2 (3)	**–**	1 (25)		1 (100)		
AML								
Relapse	6 (7)	6 (8)	**–**	**–**		**–**		
After ALL	1 (1)	**–**	**–**	1 (25)		**–**		
MDS	2 (2)	2 (3)	**–**	**–**		**–**		
JMML	2 (2)	1 (1)	1 (20)	**–**		**–**		
NHL relapse	2 (2)	2 (3)	**–**	**–**		**–**		
MLD	1 (1)	1 (1)	**–**	**–**		**–**		
HL relapse	1 (1)	1 (1)	**–**	**–**		**–**		
DBA	1 (1)	1 (1)	**–**	**–**		**–**		
Solid oncology (%)	**49** (**56)**	**43** (**56)**	**4** (**80)**	**1** (**25)**	**1** (**100)**	**–**	**1** (**100)**	**1** (**100)**
NBL	12 (13)	8 (9)	3 (60)^a^	**–**	1 (100)	**–**	1 (100)^a^	**–**
ES	15 (17)	14 (18)	**–**	1 (25)	**–**		**–**	**–**
RMS	11 (13)	11 (14)	**–**	**–**	**–**		**–**	**–**
WT	6 (7)	6 (8)	**–**	**–**	**–**		**–**	**–**
OS								
Primary	2 (2)	2 (3)	**–**	**–**	**–**		**–**	**–**
Relapse	1 (1)	**–**	1 (20)^b^	**–**	**–**		**–**	1 (100)^b^
Nongerminoma	2 (2)	2 (3)	**–**	**–**	**–**		**–**	**–**
Neuro-oncology (%)	**13** (**15)**	**13** (**17)**	**–**	**–**	**–**	**–**	**–**	**–**
Medulloblastoma	12 (14)	12 (16)	**–**	**–**	**–**	**–**	**–**	**–**
Ependymoma	1 (1)	1 (1)						
Primary malignancy (%)	75 (85)	66 (86)	4 (80)	2 (50)	1 (100)	**–**	1 (100)	**–**
Indication OTC procedure (%)								
CED	53 (60)	48 (62)	3 (60)	1 (25)	1 (100)	**–**	1 (100)	1 (100)
SCT/TBI	19 (22)	15 (19)	1 (2)	2 (50)	**–**	1 (100)	**–**	**–**
WAI	1 (1)	1 (1)	**–**	**–**	**–**	**–**	**–**	**–**
Combined indications								
CED and SCT	6 (7)	5 (6)	**–**	1 (25)	**–**	**–**	**–**	**–**
CED and WAI	7 (8)	6 (8)	1 (20)	**–**	**–**	**–**	**–**	**–**
CED and TBI	1 (1)	1 (1)	**–**	**–**	**–**	**–**	**–**	**–**
CED, SCT and WAI	1 (1)	1 (1)	**–**	**–**	**–**	**–**	**–**	**–**
Expected CED in g/m^2^ (%)								
<6.0	8 (9)	8 (11)	**–**	**–**	**–**	**–**	**–**	**–**
≥6.0	58 (67)	51 (66)	5 (100)	4 (100)	1 (100)	1 (100)	1 (100)	1 (100)
Expected radiotherapy in Gy (%)								
10–20	3 (3)	1 (1)	**–**	1 (25)	**–**	1 (100)	**–**	**–**
>20	2 (2)	2 (3)	**–**	**–**	**–**	**–**	**–**	**–**
Not reported	14 (16)	12 (16)	**–**	**–**	**–**	**–**	**–**	**–**
Laparoscopic oophorectomy circumstances								
Median age at time of LO in years (range)	6.5 (1–18)	7.0 (1–18)	2.0 (2–9)	9.5 (6–10)	1.0	6.0	2.0	9.0
Treatment naive at LO (%)	18 (20)	14 (18)	1 (20)	3 (75)	**–**	**–**	**–**	**–**
CED at time of LO in g/m^2^ (%)								
0	18 (20)	14 (18)	1 (20)	3 (75)	**–**	**–**	**–**	1 (100)
>0≤4.0	50 (57)	46 (60)	2 (40)	1 (25)	1 (100)	1 (100)	**–**	**–**
4.0–6.0	9 (10)	7 (9)	1 (20)	**–**	**–**	**–**	1 (100)	**–**
≥6.0	1 (1)	1 (1)	**–**	**–**	**–**	**–**	**–**	**–**
NA	10 (11)	9 (12)	1 (20)	**–**	**–**	**–**	**–**	
Median time between start of cancer treatment and LO in days (range)	61.0 (2–1,228)	62.0 (2–1,228)	58.0 (2–112)	75.5 (43–108)	59.0	58.0	112.0	2.0
Median time between start of last chemotherapy course and LO in days (range)	14.0 (3–457)	16.0 (3–457)	12.5 (6–21)	10.0	12.0	14.0	17.0	**–**
Platelet count before LO (%)								
≥100 × 10^9^/L	59 (67)	54 (70)	1 (20)	3 (75)	1 (100)	**–**	**–**	1 (100)
50–100 × 10^9^/L	11 (13)	9 (12)	1 (20)	1 (1)	**–**	**–**	1 (100)	**–**
≤50 × 10^9^/L^c^	10 (11)	6 (8)	3 (60)	**–**	**–**	1 (100)	**–**	**–**
Combined procedure with LO (%)	38 (43)	33 (43)	**–**	3 (75)	1 (100)	1 (100)	**–**	**–**
Antibiotic prophylaxis given before LO (%)	68 (77)	59 (77)	4 (80)	3 (75)	1 (100)	1 (100)	1 (100)	**–**
Patient in neutropenia at time of LO (%)	12 (14)	10 (13)	**–**	2 (50)	**–**	**–**	**–**	**–**
Median time between LO and postoperative chemotherapy in days (range)	1.0 (0–98)^d^	1.0 (0–98)^d^	3.0 (0–9)	1.0 (0–1)	26.0	11.0	4.0	0

*Note:* Numbers in bold depict total number of patients respectively. ALL = acute lymphoblastic leukemia; AML = acute myeloid leukemia; CED = cyclophosphamide equivalent dose; DBA = Diamond-Blackfan anemia; ES = Ewing’s sarcoma; HL = Hodgkin’s lymphoma; JMML = juvenile myelomonocytic leukemia; LO = laparoscopic oophorectomy; MDS = myelodysplastic syndrome; MLD = metachromatic leukodystrophy; n = number of patients; NA = not available; NBL = neuroblastoma; NHL = non-Hodgkin’s lymphoma; OS = osteosarcoma; OTC = ovarian tissue cryopreservation; RMS = rhabdomyosarcoma; SCT = stem cell transplantation; TBI = total body irradiation; WAI = whole abdominal irradiation; WT = Wilms tumor.

a One NBL patient with mild port site bleeding (grade 2) on day 2 and failure to wean (grade 4) on day 0 (Table 3).

b One OS patient with mild port site bleeding (grade 2) on day 2 and a port site (umbilical) abscess (grade 3) on day 25 (Table 3).

c All patients with a platelet count <50 × 10^9^/L were given platelet transfusions before the oophorectomy procedure.

d One patient had started her first chemotherapy course 98 days after oophorectomy, radiotherapy was started 4 weeks postoperatively.

### Characteristics and settings of patients with and without any oophorectomy-related complications

Patients who had experienced any complication related to oophorectomy had a median age at diagnosis of 4.5 years (range 1–14 years), whereas patients without complications had a median age of 7.0 years (range 0–18 years) at diagnosis (Table 2). Median age at time of oophorectomy was 6.0 years (range 1–14 years) and 7.0 years (range 1–18 years), respectively, for patients with and without complications. Patients with oophorectomy-related complications were either diagnosed with hemato-oncological (n = 5/11, 45%) or solid (n = 6/11, 55%) tumors (Table 2), whereas the patients without complications consisted of 21/77 (27%) hemato-oncological, 43/77 (56%) solid tumor, and 13/77 (17%) neuro-oncological patients. Out of 11 patients with complications, 9 (82%) had a primary malignancy, whereas the noncomplication subset consisted of 66/77 (86%) patients with a primary malignancy. Four out of 18 (22%) patients who had not received chemotherapy before oophorectomy, experienced complications. Seven out of 70 (10%) patients who had received chemotherapy before oophorectomy, experienced complications. The median time between the last chemotherapy before oophorectomy procedure was 12.0 days (range 6–21 days) for those with complications and 16.0 days (range 3–457 days) for patients without complications. Combined procedures had been performed in 5/11 (45%) of the patients with complications and in 33/77 (43%) of the patients without complications. In both groups, the next chemotherapy course after the oophorectomy procedure had been started after a median time of 1.0 day (range 0–98 days). The used trocar size was 5 millimeters in all patients.

### Description of CD grade 3 and 4 LO-related complications

Serious complications were registered in 3 patients, including 2 patients who experienced CD grade 4 complications, and 1 who experienced a CD grade 3 complication (Table 3). Patients who experienced CD grade 4 complications included 1 relapsed acute lymphoblastic leukemia (ALL) patient (age 6 years, patient 1 [Table 3]) who experienced endotracheal tube obstruction by hemorrhagic fluid after oophorectomy, after a preceding BAL during the same anesthesia as the oophorectomy, leading to bradycardia. Bradycardia occurred again on day 6 at the time of a central line infection (CD grade 2), of unknown origin. Both occasions led to a resuscitation setting, for which no chest compressions, but only inotropes and vasopressors were necessary, and both resuscitations were successful. In the same patient, this line infection and acute event on day 6 preceded a period of reversible acute kidney insufficiency (CD grade 2) and subsequently multiorgan failure on day 10. After 32 days, she was discharged from the PICU. The second grade 4 complication was in an abdominal neuroblastoma patient (age 2 years, patient 2 [Table 3]), who needed prolonged postoperative mechanical ventilation and (for 48 hours) admission to the PICU after surgery, due to failure to wean from mechanical ventilation, after bronchospasms during anesthetic induction. This patient also experienced CD grade 2 mild port site bleeding on the second day after surgery, for which she received a platelet transfusion, after which no further bleeding occurred. Both patients are currently alive and in continuous complete remission at the last moment of follow-up (follow-up time is 3 and 1 years, respectively).

**Table 3:** Characteristics of childhood cancer patients with laparoscopic oophorectomy-related complications (November 2020–April 2024).

Patient	Age at diagnosis (y)	Diagnosis	Start chemotherapy prior LO (d)^a^	Preoperative condition						Combined procedure	Complication					Outcome
				Blood count				PEWS	ABx prophylaxis for LO		Type of complication	Day	CD	Intervention	Start chemo after LO (days)^b^	
				Hb	Plt	PTx	WBC									
1	6	ALL 1st relapse	14	5.4	35	Yes	NA	0	Cefazolin	BAL^c^	Endotracheal tube obstruction → bradycardia, after BAL^d^	0^e^	4	Resuscitation, PICU(32, V+)	11	Alive in remission (LF 4 yr. 4 mo.)
											CLABSI →	3^f^	2	ABx		
											AKI →	6^f^	2	Dialysis		
											Bradycardia →	6^f^	4	Resuscitation		
											Multi organ failure	10^g^	4	PICU extended		
2	2	NBL	17	NA	65	No	22.3	0	Cefazolin	No	Failure to wean	0^h^	4	PICU (2, V+)	4	Ongoing treatment (LF 1 yr. 7 mo)
											Port site (umbilical) bleeding	2^f^	2	RBC transfusion		
3	9	OS 2nd relapse	None	NA	216	No	12.4	0	No	No	Port site (umbilical) bleeding	0^h^	2	RBC transfusion	0	Deceased 6 mo. postdiagnosis of PD
											Port site (umbilical) abscess	25^g^	3	Re-operation		
4	2	NBL	6	4.8	32	Yes	5.1	0	Cefazolin	No	Port site (lateral) bleeding	0^e^	2	2nd Plt transfusion	2	Deceased 1 yr. postdiagnosis of PD
5	3	JMML	21	NA	10	Yes^i^	4.0	0	Cefazolin	No	Port site (umbilical) bleeding	1^j^	2	RBC and Plt transfusion	3	Ongoing treatment after relapse (LF 3 yr. 7 mo)
6	2	NBL	8	NA	7	Yes	5.8	0	Cefazolin	No	Port site (umbilical) bleeding	1^j^	2	RBC transfusion	9	Deceased 6 mo. postdiagnosis of PD
7	9	AML after ALL	None	NA	69	Yes	2.5^k^	1^l^	Cefazolin	Hickman, LP	Port site (umbilical) infection	2^f^	2	ABx	0	Deceased 1 yr. postdiagnosis of PD
8	6	ALL	10	NA	168	No	1.9^j^	0	Cefazolin	BMB, LP	Port site (umbilical) infection	2^f^	2	ABx	1	Alive in remission (LF 5 yr. 7 mo)
9	14	ES	None	7.2	390	No	6.2	0	Cefazolin	PAC, BMB	Port site (umbilical) infection	17^g^	2	ABx	1	Alive in remission (LF 4 yr. 5 mo)
10	10	ALL 1st relapse	None	NA	140	No	2.7	0	No	No	Port site (umbilical) infection	23^g^	2	ABx	1	Alive in remission (LF 5 yr. 3 mo)
11	1	NBL	12	NA	110	No	1.9	0	Cefazolin	PAC, apheresis	Desaturation	0^h^	2	O2, ENT consult, dexamethasone	26	Deceased 5 mo. postdiagnosis of PD

ABx = antibiotics; AKI = acute kidney insufficiency; ALL = acute lymphoid leukemia; AML = acute myeloid leukemia; BAL = bronchoalveolar lavage; BMB = bone marrow biopsy; CD = Clavien Dindo classification grade; CLABSI = central line-associated bloodstream infection; ENT = ear nose throat specialist; ES = Ewing’s sarcoma; Hb = hemoglobin (x10^9^/L); JMML = juvenile myelomonocytic leukemia; LF = time between oophorectomy and last follow-up; LO = laparoscopic oophorectomy; LP = lumbar puncture; mo.: month; MOF = multiorgan failure; NA = not available; NBL = neuroblastoma; O2 = noninvasive oxygen suppletion; OS = osteosarcoma; Plt = platelets (x10^9^/L); PAC = port-a-cath; PD = progressive disease; PEWS = pediatric early warning signs; PICU = pediatric intensive care unit; PICU(n) = n is days admitted to PICU; V+ = invasive ventilation during PICU admission; PTx = patient received (yes/no) platelet transfusion before or during oophorectomy procedure; RBC = red blood cell; WBC = white blood cells (x10^9^/L); yr. = year.

a Days since last chemotherapy before oophorectomy, None meaning patients had not received any chemotherapy for current malignancy before oophorectomy.

b Days between oophorectomy and start of next chemotherapy.

c Laparoscopic oophorectomy, BAL, tooth extraction, and triple lumen planned. After resuscitation after BAL and oophorectomy procedure performed, but tooth extraction and triple lumen procedures omitted.

d Bradycardia caused by obstruction of endotracheal tube by hemorrhagic fluid.

e Day 0 during oophorectomy procedure, prior to extubation during anesthesia.

f Day 2–7 after oophorectomy procedure.

g Day 8–30 after oophorectomy procedure.

h Day 0 after oophorectomy procedure, within 24 hours after extubation.

i Two platelet infusions.

j Day 1 after oophorectomy procedure, 24 hours after extubation.

k Patient in neutropenia at time of laparoscopic oophorectomy.

l PEWS of 1 because of elevated heart rate due to stress, no signs of infection before oophorectomy procedure.

One relapsed osteosarcoma patient (age 9 years, patient 3 [Table 3]) developed a CD grade 3 complication, i.e., port site (umbilical) abscess on day 25 after LO. She had experienced mild port site bleeding (CD grade 2) within 24 hours after oophorectomy and had started with the next chemotherapy course directly postoperatively. She needed surgical decompression of the port site abscess and antibiotic treatment (day 25), after which she fully recovered. Unfortunately, she passed away 6 months later due to the progression of her cancer.

### Description of CD grade 2 LO-related complications

Clavien Dindo grade 2 complications were registered in 8 patients, in addition to the CD grade 2 complications registered in the 3 patients described above (Table 3). Mild port site bleeding (CD grade 2) occurred in 3 additional patients, diagnosed with neuroblastoma (n = 2) and juvenile myelomonocytic leukemia (n = 1) (Table 3), of which 1 occurred during the oophorectomy procedure and 2 within 2 days postoperatively. Four out of the total 5 patients (80%) with mild port site bleeding, had a platelet count below 100 × 10^9^/L (3 [60%] had a count below 50 × 10^9^/L). In patients who did not experience bleeding postoperatively, 7 out of 83 (8%) had a platelet count below 50 × 10^9^/L. All patients with a platelet count below 50 × 10^9^/L had received elective platelet transfusions before OTC procedure. Those with mild port site bleeding received additional platelet (and red blood cell) transfusions, and no further bleeding occurred (Table 3).

In addition to the patient mentioned earlier who developed a port site abscess on day 25, 4 more patients (5%), diagnosed with ALL (n = 2), acute myeloid leukemia (AML) (n = 1), and Ewing’s sarcoma (n = 1), had developed a CD grade 2 port site (umbilical) infection (Table 3). Two (50%) were reported 2 days after oophorectomy, the other 2 (50%) in the 3rd week (after the next chemotherapy course, followed directly after oophorectomy). Three out of these 4 patients (75%) had received preoperative antibiotic prophylaxis before the procedure, and 2 (50%) were neutropenic during the procedure. Neither of the neutropenic patients had received granulocyte colony-stimulating factor. All 4 patients had started the next chemotherapy course on the same or the next day after oophorectomy. All 4 patients recovered after intravenous antibiotic treatment. Preoperative antibiotic prophylaxis had been administered in 4 out of 5 (80%) patients with port site infections and in 64 out of 83 (77%) patients who did not develop port site infections. Two out of 5 (40%) patients with port site infections were neutropenic at the time of LO, vs. 10 out of 83 (12%) of patients without port site infections.

One patient (1%), diagnosed with abdominal neuroblastoma, experienced CD grade 2 desaturations within 24 hours after the oophorectomy procedure (Table 3). Like all other patients with complications, she had a normal PEWS and blood count before the procedure, and the last chemotherapy course had been started more than a week before LO. She needed oxygen supplementation as well as dexamethasone because of a mild stridor after intubation and administration of anesthetic agents. The patient rapidly recovered and admission to the PICU was not necessary.

### Overall outcome

Out of 88 patients who underwent LO, 11 patients died (13%). Median time of death was 5.5 months after oophorectomy (range 2–71 months). Eight patients (73%) died of progressive disease, 2 (18%) after relapse, and 1 (9%) of multiorgan failure after sepsis (64 days after oophorectomy), after myeloablative therapy and stem cell transplantation (Supplemental Fig. 1, available online). None of the deaths were related to or caused by a direct complication of oophorectomy.

## Discussion

The objective of our study was to systematically and critically appraise the safety of the LO procedure in the relatively *novel* setting of children with cancer, utilizing a prospective national cohort. This safety evaluation is important because during the last decade this procedure has become internationally considered to be standard of care in children with cancer, who will receive gonadotoxic treatment (9, 10). Our study shows that 87% of patients (n = 77/88) experienced no complications, 3 out of 88 patients (3.4%) experienced serious complications (1 CD grade 3, 2 CD grade 4) and 9% of patients (8/88) experienced minor complications (CD grade 2). This frequency is in line with that in reports in (young) adults (Supplemental Table 3). Still, although none of the 88 patients died due to complications of the procedure, this frequency needs to be taken seriously, as LO is an elective procedure, and it is pursued in seriously ill children. This is underscored by the 8 patients who experienced CD grade 2 complications, with the most common complications of port site bleeding (6%) and infection (6%), which seems high in comparison to available reports (Table 4, Supplemental Table 3) (22, 23, 24, 25, 26, 27, 28, 29, 30, 31, 32, 33, 34). This could be due to the fact that grade 2 complications have not been registered systematically in the past. Also, the current study is the first that aimed to capture such events in a national prospective cohort of pediatric cancer patients who undergo LO. Furthermore, this is the only study that used CD grading. Hence, the complication rates in existing literature may be tampered by registration bias (13, 14, 15, 16, 17, 18, 19, 20, 21, 22, 23, 24, 25, 26, 27, 28, 29, 30, 31, 32, 33, 34). We value all complications, even grade 2 complications such as port site infections and bleeding, as relevant in children with cancer because they may induce serious conditions in immunocompromised children and cause undesired delay of oncological treatment.

**Table 4:** Current available literature on the safety of laparoscopic ovarian tissue cryopreservation procedure in children.

Paper	Study design	Sample size n (n∗)	Median age at OTC in years^a^ (range in years)	Indication OTC	Method of OTC	Patients with complications		OTC-related mortality	Not OTC-related mortality, including cause of death
						CD grade 1–2	CD grade 3–4		
Bath et al., 2004 (24)	Case report	1	14.9	Cancer	Ovarian cortex biopsies	None	None	n = 0	n = 0
Poirot et al., 2007 (32)	Prospective cohort	47	5 (0.8–15)	Cancer	Unilateral complete oophorectomy	None	None	n = 0	PD (n = 10)
Revel et al., 2009 (33)	Prospective cohort	19	15 (5–20)	Cancer (n = 17), nonmalignant disease (n = 2)	Unilateral oophorectomy	None	None	n = 0	PD (n = 2), treatment related (n = 2)
Jadoul et al., 2010 (28)	Prospective cohort	58	Mean 10.4 years (0.8–15.8)	Cancer (n = 48), nonmalignant disease (n = 10)	Unilateral complete oophorectomy (n = 20), cortical biopsies (n = 38)	None	None	n = 0	Disease related^b^ (n = 8)
Babayev et al., 2013 (23)	Prospective observational cohort	28 (NA)	14.6 (2.3–21.0)	Cancer (n = 21), nonmalignant disease (n = 7)	Unilateral complete oophorectomy	None	None	n = 0	NA
Lima et al., 2014 (30)	Prospective cohort	54	Mean 13.4 years (NA)	Cancer (n = 47), nonmalignant disease (n = 7)	Unilateral partial (2/3) oophorectomy	Mild hemorrhage (NA)	None	n = 0	NA
Biasin et al., 2015 (25)	Prospective cohort	47	13 (2.7–20.3)	Cancer (n = 40), nonmalignant disease (n = 7)	Ovarian cortex biopsies	None	None	n = 0	PD (n = 7)
Abir et al., 2016 (22)	Prospective cohort	42	Mean 13 years (2–18)	Cancer (n = 40), nonmalignant disease (n = 2)	Unilateral complete (n = 27) or partial (n = 15) oophorectomy	None	None	n = 0	NA
Chambon et al., 2016 (27)	Retrospective cohort	36	13 (2–19)	Cancer (n = 28), nonmalignant disease (n = 8)	Bilateral partial (1/3) oophorectomy	None	Hemorrhage with re-operation (**n = 1**)	n = 0	n = 10^b^
Rowell et al., 2019 (34)	Retrospective cohort	64 (NA)	12 (0.4–23)	Cancer (n = 53), nonmalignant disease (n = 11)	Unilateral complete oophorectomy	None	None	n = 0	Disease related^b^ (n = 6)
Lotz et al., 2020 (31)	Retrospective survey cohort	53	Mean 14.8 years (6–17)	Cancer (n = 40), nonmalignant disease (n = 13)	Unilateral partial (2/3) oophorectomy	None	Hemorrhage with re-operation (**n = 1**)	n = 0	n = 12^b^
Brodigan et al., 2021 (26)	Retrospective cohort	13 (12)	13 (1–22)	Cancer (n = 7), nonmalignant disease (n = 6)	Unilateral complete oophorectomy with fallopian tube preservation	Fever without aplasia (n = 1)	None	n = 0	NA
Kondo et al., 2025 (29)	Retrospective survey cohort	110	Mean 8.9 years (4.9–13.9)	Cancer (n = 89), nonmalignant disease (n = 21)	Unilateral complete oophorectomy	Postoperative infection (n = 1)	None	n = 0	NA

CD = Clavien Dindo classification grade; n = number of patients; n∗ = subset of sample size with age <19 years in studies with patients aged >18 years; NA = not available; OTC = ovarian tissue cryopreservation; PD = progressive disease.

a Unless otherwise specified.

b Not further specified in papers, other than that death was not related to OTC procedure.

The correlation between the severity of complications and the clinical condition using preceding PEWS monitoring is suboptimal. We also could not identify other specific risk factors for the occurrence of CD grade 3 and 4, nor for grade 2 complications. In general, the conditions under which the procedure had been performed were similar in the patients with and without complications. It may be argued that a rapid start of chemotherapy after oophorectomy may be a risk factor for the occurrence of the 5 port site infections that we observed after surgery, i.e., a certain window for wound recovery may be reasonable, if clinically acceptable. The clinical status as a determinant for complications has also been assessed in other available studies with similar frequencies of complications (13, 14, 15, 16, 17, 18, 19, 20, 21, 22, 23, 24, 25, 26, 27, 28, 29, 30, 31, 32, 33, 34), which aimed to analyze the safety of LO in patients with cancer. These studies included both children and adult series, with ages ranging between 1 and 44 at the time of surgery (Table 4, Supplemental Table 3). We could not find available literature on the safety of LO outside the context of fertility preservation in patients with cancer.

So far, only 1 death related to an elective oophorectomy procedure has been reported by Imbert et al. (16) in a 26-year-old woman diagnosed with systemic lupus erythematosus that was planned to undergo gonadotoxic immunosuppressive treatment (out of a cohort of 255 patients, age range not reported), who experienced severe buccal and nasal hemorrhage after anesthesia and extubation (Supplemental Table 3). She was admitted to the intensive care unit, developed acute respiratory distress syndrome and died 7 days postoperatively from sepsis. This may illustrate, similar to the resuscitated patient described in our study, the serious need to estimate the risk of, and find ways to avoid potential respiratory complications during the oophorectomy procedure, especially as half of our procedures were combined with other interventions under anesthesia. Nine heterogeneous cohorts reported a total number of 115 deaths combined (16, 18, 27, 28, 31, 32, 34), but none of these deaths were considered to be related to the oophorectomy, but to the disease and/or its treatment, without further specification (Table 4, Supplemental Table 3). In our cohort of 88 pediatric patients, the first and largest study on a national cohort, we report 11 deaths, none of which are considered to be related to the OTC procedure. One patient died 2 months after the oophorectomy procedure. This patient, aged 16 years, was diagnosed with myelodysplastic syndrome and was scheduled to undergo HSCT, including myeloablative therapy 9 days after the OTC procedure (Supplemental Fig. 1). She developed a candidemia sepsis 12 days after HSCT (21 days postoperatively), and was admitted to the PICU 24 days postoperatively, where she passed away from multiorgan failure and sepsis 40 days after PICU admission (64 days postoperatively). The port sites had healed well, and there were no signs of a site infection in the postoperative period. Before the OTC procedure and the start of myeloablative therapy, she had been in good condition, despite experiencing recurrent fever. The myeloablative therapy before her HSCT, rather than the oophorectomy procedure, was considered to be the main contributor to her deterioration and course of her clinical condition. This case is described in detail in Supplemental Figure 1.

We observed in total 5 (6%) CD grade 2 port site bleeding (Table 3). Four out of these 5 patients (80%) had platelet counts below 100 × 10^9^/L (3 below 50 × 10^9^/L, 60%) preoperatively, compared with 20% (n = 17/83) of patients who did not experience port site bleeding. Chambon et al. (27), Lotz et al. (31), and Perelli et al. (19) reported that out of a total of 3 cohorts of in total 400 patients (children and adults) combined, 4 patients (1%, ages not reported, no grading used) experienced hemorrhages, that required a second operation (Table 4, Supplemental Table 3). Lima et al. (30) and Rodriguez-Wallberg et al. (20) reported that mild hemorrhage occurred in their cohorts; however, they did not specify in how many patients this occurred nor how severe these hemorrhages were. In none of the studies, it was further specified for what diagnoses individual patients were treated, what interventions were performed besides reoperations or admission to an intensive care unit, nor what the extent of the bleeding was (Table 4, Supplemental Table 3).

Five (6%) of our patients experienced port site (umbilical) infections (1 CD grade 3, 4 CD grade 2), of which 1 needed surgery at day 25 (Table 3). Rosendahl et al. (21) reported that 2 out of 92 patients (2%, aged 9 and 37 years) developed a port site infection, for which they needed a second operation (Supplemental Table 3). Kondo et al. (29) reported that 1 out of 110 patients (1%) developed a postoperative infection, which was not further specified by the authors, and for which she received antibiotic treatment (Table 4). No other studies reported data on port site infections postoperatively. In the reported cases, again, grading was not taken into account, and therefore results are difficult to compare.

In our cohort, 3 out of 88 patients experienced serious complications. We could not identify a common denominator that could predict the occurrence of complications. In general, we try to plan the optimal moment to pursue elective LO after confirming adequate vital status, guaranteeing adequate platelet counts (>50 × 10^9^/L) (44), avoiding surgery in patients with neutropenia, as well as avoiding concomitant burdensome elective procedures. Complication rates in patients who underwent combined procedures (13%) were similar to those reported in patients who underwent stand-alone procedures (12%). The use of antibiotic prophylaxis is not standard for LO, according to national guidelines (41). In our cohort, 68 patients (77%) received antibiotic prophylaxis. Most of these patients underwent a combined procedure, and an additional 5 patients received antibiotics, of which we anticipate they may have received this because of neutropenia at the time of the procedure. We learned that we may avoid combining intubation for the oophorectomy procedure and bronchoalveolar lavage (BAL), if possible, especially in children with thrombocytopenia, to avoid possible respiratory complications. One could argue whether the complications after BAL and oophorectomy in one of our patients (patient 1, Table 3) may indeed have been related to the BAL rather than the LO, but as this is not certain, we intended to be transparent in our descriptive study, and that is why this case is included in our cohort. Although we regard chemo-naive status as ideal for oophorectomy, at our center LO is delayed in specific situations, such as in large abdominal tumors, where, before starting chemotherapy, it is sometimes not possible to safely remove the ovary. Likewise, in acute leukemia patients, LO is postponed for 6 to 8 weeks after the start of cancer treatment, to avoid the occurrence of leukemic cells in the cryopreserved cortex tissue as much as possible. Future evaluations need to show whether a slight delay of chemotherapy postoperatively may decrease the number of port site infections in these patients and whether this will influence results after auto-transplantation at a later stage in life. Our study underscores the importance of careful selection of patients for OTC and for pursuing oophorectomy in a controlled, centralized environment, with highly equipped pediatric gynecological, surgical-oncological, and anesthetic expertise, the availability of a PICU, embedded in a dedicated oncofertility team.

The strength of this study is that it provides the first evaluation of the safety of LO procedures in pediatric cancer patients based on a national prospective cohort. However, as this pediatric cancer cohort is relatively small and heterogeneous (n = 88), it did not allow for any statistical analyses to identify independent contributing risk factors for the occurrence of complications. Besides, we realize that most patients in our cohort (80%) had received chemotherapeutic, and in some cases mild gonadotoxic, exposure before LO. This may not apply to other centers worldwide that perform most procedures before gonadotoxic exposure only. More information on the safety of LO in childhood cancer patients is therefore necessary, preferably from prospective registries in larger international cohorts, to further explore the risks and benefits of this procedure.

## Conclusion

We conclude that LO for OTC is a relatively safe procedure to preserve future fertility options for girls with cancer with a high risk of gonadal failure and consequent future infertility. However, LO can be accompanied by complications in children with cancer. Therefore, careful stratification, safety monitoring and identification of risk factors for complications based on larger cohorts in future studies are needed. Prevention of complications of LO is an important obligation to balance the future benefits against the risks of LO to enhance the future chance of pregnancy for girls with cancer.

## CRediT Authorship Contribution Statement

**Nikita H.Z. Clasen:** Writing – review & editing, Writing – original draft, Visualization, Validation, Software, Resources, Project administration, Methodology, Investigation, Formal analysis, Data curation, Conceptualization. **M.E. Madeleine van der Perk:** Writing – review & editing, Methodology, Investigation, Formal analysis, Data curation, Conceptualization. **Marianne D. van de Wetering:** Writing – review & editing, Supervision. **Annelies M.E. Bos:** Writing – review & editing. **Anne-Lotte L.F. van der Kooi:** Writing – review & editing. **Kim van Loon:** Writing – review & editing. **Margreet A. Veening:** Writing – review & editing. **Irene M. IJgosse:** Writing – review & editing. **Sebastian J.C.M.M. Neggers:** Writing – review & editing. **Martine van Grotel:** Writing – review & editing. **Dorine Bresters:** Writing – review & editing. **Hanneke M. van Santen:** Writing – review & editing. **Willem M.J.A. Verpoest:** Writing – review & editing. **Leendert H.J. Looijenga:** Writing – review & editing. **Brigitte Arends:** Writing – review & editing. **Jeanette van Leeuwen:** Writing – review & editing. **Simone L. Broer:** Writing – review & editing. **Alida F.W. van der Steeg:** Writing – review & editing, Supervision, Methodology, Investigation, Formal analysis, Data curation, Conceptualization. **Marry M. van den Heuvel-Eibrink:** Writing – review & editing, Writing – original draft, Visualization, Validation, Supervision, Software, Resources, Project administration, Methodology, Investigation, Funding acquisition, Formal analysis, Data curation, Conceptualization.

## Declaration of Interests

N.H.Z.C. reports funding from Princess Máxima Center Foundation and EU TREL & SCARLET for the submitted work. M.E.M.v.d.P. reports funding from Princess Máxima Center Foundation, Child Health Boost grant 2017, and Stichting Kinderoncologisch Centrum Rotterdam (sKOCR) for the submitted work. M.D.v.d.W. has nothing to disclose. A.M.E.B. has nothing to disclose. A-L.L.F.v.d.K. reports payments received for lectures etcetera are directly paid out to employer, the Leids University Medical Center; ESHRE Campus facilitates costs for meetings and travel for speakers and moderators. K.v.L. reports funding from H2023 MSCA DN (EU) Beneficiary in SMARTTEST project outside the submitted work; travel support Attending/Keynote speaker 3SCTS Sydney. M.A.V. has nothing to disclose. I.M.I. has nothing to disclose. S.J.C.M.M.N. has nothing to disclose. M.v.G. has nothing to disclose. D.B. has nothing to disclose. H.M.v.S. reports funding from Investigator initiated grant Rhythm outside the submitted work; Payment for lecture 2025 Rhythm; payment for travel costs by Rhythm. W.M.J.A.V. reports funding from Research Grant Merck B.V. and Research Grant IBSA outside the submitted work; consulting fees CooperSurgical and Cook Medical; Advisory Board Goodlife. L.H.J.L. reports funding from KiKa Foundation outside the submitted work. B.A. has nothing to disclose. J.v.L. has nothing to disclose. S.L.B. reports funding from ZonMW and Besins Healthcare outside the submitted work. A.F.W.v.d.S. is Secretary quality and safety of the executive board of the Netherlands Society for Surgery. M.M.v.d.H-E. has nothing to disclose.
