# Supplementary material for: Safety of laparoscopic oophorectomy in a Dutch national pediatric cancer cohort
Source: F S Rep. 2025 Dec 2;7(1):69–80. doi: 10.1016/j.xfre.2025.11.008 (PMC12905605; doi:10.1016/j.xfre.2025.11.008)
Supplement: Supplementary Materials [file mmc1.docx]

**Supplemental Table 1. Current available literature on pregnancy and live birth rate after auto-transplantation of cryopreserved ovarian tissue in (young) adult women after cancer**

| 1. Demeestere I, Simon P, Emiliani S, Delbaere A, Englert Y. Fertility preservation: successful transplantation of cryopreserved ovarian tissue in a young patient previously treated for Hodgkin's disease. Oncologist. 2007;12:1437-42. 2. Dittrich R, Lotz L, Keck G, Hoffmann I, Mueller A, Beckmann MW, et al. Live birth after ovarian tissue autotransplantation following overnight transportation before cryopreservation. Fertil Steril. 2012;97:387-90. 3. Dittrich R, Hackl J, Lotz L, Hoffmann I, Beckmann MW. Pregnancies and live births after 20 transplantations of cryopreserved ovarian tissue in a single center. Fertil Steril. 2015;103:462-8. 4. Donnez J, Dolmans MM, Demylle D, Jadoul P, Pirard C, Squifflet J, et al. Livebirth after orthotopic transplantation of cryopreserved ovarian tissue. Lancet. 2004;364:1405-10. 5. Donnez J, Dolmans MM, Pellicer A, Diaz-Garcia C, Sanchez Serrano M, Schmidt KT, et al. Restoration of ovarian activity and pregnancy after transplantation of cryopreserved ovarian tissue: a review of 60 cases of reimplantation. Fertil Steril. 2013;99:1503-13. 6. Dueholm Hjorth IM, Kristensen SG, Dueholm M, Humaidan P. Reproductive outcomes after in vitro fertilization treatment in a cohort of Danish women transplanted with cryopreserved ovarian tissue. Fertil Steril. 2020;114:379-87. 7. Dunlop CE, Brady BM, McLaughlin M, Telfer EE, White J, Cowie F, et al. Re-implantation of cryopreserved ovarian cortex resulting in restoration of ovarian function, natural conception and successful pregnancy after haematopoietic stem cell transplantation for Wilms tumour. J Assist Reprod Genet. 2016;33:1615-20. 8. Ernst E, Bergholdt S, Jorgensen JS, Andersen CY. The first woman to give birth to two children following transplantation of frozen/thawed ovarian tissue. Hum Reprod. 2010;25:1280-1. 9. Fabbri R, Vicenti R, Magnani V, Paradisi R, De Meis L, Raimondo D, et al. Ovarian tissue transplantation: 10 years of experience at the Bologna University. Front Endocrinol (Lausanne). 2024;15:1332673. 10. Hoekman EJ, Louwe LA, Rooijers M, van der Westerlaken LAJ, Klijn NF, Pilgram GSK, et al. Ovarian tissue cryopreservation: Low usage rates and high live-birth rate after transplantation. Acta Obstet Gynecol Scand. 2020;99:213-21. 11. Jensen AK, Kristensen SG, Macklon KT, Jeppesen JV, Fedder J, Ernst E, et al. Outcomes of transplantations of cryopreserved ovarian tissue to 41 women in Denmark. Hum Reprod. 2015;30:2838-45. 12. Khattak H, Malhas R, Craciunas L, Afifi Y, Amorim CA, Fishel S, et al. Fresh and cryopreserved ovarian tissue transplantation for preserving reproductive and endocrine function: a systematic review and individual patient data meta-analysis. Hum Reprod Update. 2022;28:400-16. 13. Lee JR, Lee D, Park S, Paik EC, Kim SK, Jee BC, et al. Successful in Vitro Fertilization and Embryo Transfer after Transplantation of Cryopreserved Ovarian Tissue: Report of the First Korean Case. J Korean Med Sci. 2018;33:e156. 14. Meirow D, Ra'anani H, Shapira M, Brenghausen M, Derech Chaim S, Aviel-Ronen S, et al. Transplantations of frozen-thawed ovarian tissue demonstrate high reproductive performance and the need to revise restrictive criteria. Fertil Steril. 2016;106:467-74. 15. Muller A, Keller K, Wacker J, Dittrich R, Keck G, Montag M, et al. Retransplantation of cryopreserved ovarian tissue: the first live birth in Germany. Dtsch Arztebl Int. 2012;109:8-13. 16. Oktay K. Spontaneous conceptions and live birth after heterotopic ovarian transplantation: is there a germline stem cell connection? Hum Reprod. 2006;21:1345-8. 17. Rodriguez-Wallberg KA, Karlstrom PO, Rezapour M, Castellanos E, Hreinsson J, Rasmussen C, et al. Full-term newborn after repeated ovarian tissue transplants in a patient treated for Ewing sarcoma by sterilizing pelvic irradiation and chemotherapy. Acta Obstet Gynecol Scand. 2015;94:324-8. 18. Sanger N, John J, Einenkel R, Schallmoser A. First report on successful delivery after retransplantation of vitrified, rapid warmed ovarian tissue in Europe. Reprod Biomed Online. 2024;49:103940. 19. Sheshpari S, Shahnazi M, Mobarak H, Ahmadian S, Bedate AM, Nariman-Saleh-Fam Z, et al. Ovarian function and reproductive outcome after ovarian tissue transplantation: a systematic review. J Transl Med. 2019;17:396. 20. Stern CJ, Gook D, Hale LG, Agresta F, Oldham J, Rozen G, et al. First reported clinical pregnancy following heterotopic grafting of cryopreserved ovarian tissue in a woman after a bilateral oophorectomy. Hum Reprod. 2013;28:2996-9. 21. Tammiste T, Kask K, Padrik P, Idla K, Rosenstein K, Jatsenko T, et al. A case report and follow-up of the first live birth after heterotopic transplantation of cryopreserved ovarian tissue in Eastern Europe. BMC Womens Health. 2019;19:65. 22. Tanbo T, Greggains G, Storeng R, Busund B, Langebrekke A, Fedorcsak P. Autotransplantation of cryopreserved ovarian tissue after treatment for malignant disease - the first Norwegian results. Acta Obstet Gynecol Scand. 2015;94:937-41. 23. Van der Ven H, Liebenthron J, Beckmann M, Toth B, Korell M, Krussel J, et al. Ninety-five orthotopic transplantations in 74 women of ovarian tissue after cytotoxic treatment in a fertility preservation network: tissue activity, pregnancy and delivery rates. Hum Reprod. 2016;31:2031-41. 24. Andersen ST, Pors SE, Poulsen LC, Colmorn LB, Macklon KT, Ernst E, et al. Ovarian stimulation and assisted reproductive technology outcomes in women transplanted with cryopreserved ovarian tissue: a systematic review. Fertil Steril. 2019;112:908-21. 25. Dolmans MM, von Wolff M, Poirot C, Diaz-Garcia C, Cacciottola L, Boissel N, et al. Transplantation of cryopreserved ovarian tissue in a series of 285 women: a review of five leading European centers. Fertil Steril. 2021;115:1102-15. 26. Colmorn LB, Pedersen AT, Larsen EC, Hansen AS, Rosendahl M, Andersen CY, et al. Reproductive and Endocrine Outcomes in a Cohort of Danish Women following Auto-Transplantation of Frozen/Thawed Ovarian Tissue from a Single Center. Cancers (Basel). 2022;14. 27. Fabbri R, Vicenti R, Magnani V, Paradisi R, Lima M, De Meis L, et al. Ovarian tissue cryopreservation and transplantation: 20 years experience in Bologna University. Front Endocrinol (Lausanne). 2022;13:1035109. 28. Fraison E, Huberlant S, Labrune E, Cavalieri M, Montagut M, Brugnon F, et al. Live birth rate after female fertility preservation for cancer or haematopoietic stem cell transplantation: a systematic review and meta-analysis of the three main techniques; embryo, oocyte and ovarian tissue cryopreservation. Hum Reprod. 2023;38:489-502. 29. Gellert SE, Pors SE, Kristensen SG, Bay-Bjorn AM, Ernst E, Yding Andersen C. Transplantation of frozen-thawed ovarian tissue: an update on worldwide activity published in peer-reviewed papers and on the Danish cohort. J Assist Reprod Genet. 2018;35:561-70. 30. Lotz L, Bender-Liebenthron J, Dittrich R, Haberle L, Beckmann MW, Germeyer A, et al. Determinants of transplantation success with cryopreserved ovarian tissue: data from 196 women of the FertiPROTEKT network. Hum Reprod. 2022;37:2787-96. 31. Shapira M, Raanani H, Barshack I, Amariglio N, Derech-Haim S, Marciano MN, et al. First delivery in a leukemia survivor after transplantation of cryopreserved ovarian tissue, evaluated for leukemia cells contamination. Fertil Steril. 2018;109:48-53. 32. Shapira M, Dolmans MM, Silber S, Meirow D. Evaluation of ovarian tissue transplantation: results from three clinical centers. Fertil Steril. 2020;114:388-97. |
| --- |

**Supplemental Table 2. Paediatric early warning score (PEWS) as implemented in the Princess Máxima Center for Pediatric Oncology (1)**

| **Age 0 – 3 months** | | | | | | | |
| --- | --- | --- | --- | --- | --- | --- | --- |
| **Score** | **4** | **2** | **1** | **0** | **1** | **2** | **4** |
| Respiratory rate/min | <15 | 15-19 | 20-29 | 30-60 | 61-80 | 81-90 | >90 |
| Work of breathing* |  |  |  | Normal | Minimally elevated | Mildly elevated | Severely elevated and/or apnea |
| Saturation |  | <91% | 91-94% | >94% |  |  |  |
| Oxygen suppletion |  |  |  | Room air |  | Suppletion | NRB-mask/high flow O2 |
| Heart rate/min | <80 | 80-89 | 90-109 | 110-150 | 151-180 | 181-190 | >190 |
| Capillary refill time (sternal) |  |  |  | <3 seconds |  |  | ≥3 seconds |
| Blood pressure (systolic) (mmHg) | <45 | 45-49 | 50-59 | 60-80 | 81-100 | 101-130 | >130 |

*Elevated work of breathing: nasal flaring, retractions i.e. intercostal

| **Age 3 months – 1 year** | | | | | | | |
| --- | --- | --- | --- | --- | --- | --- | --- |
| **Score** | **4** | **2** | **1** | **0** | **1** | **2** | **4** |
| Respiratory rate/min | <15 | 15-19 | 20-24 | 25-50 | 51-70 | 71-80 | >80 |
| Work of breathing* |  |  |  | Normal | Minimally elevated | Mildly elevated | Severely elevated and/or apnea |
| Saturation |  | <91% | 91-94% | >94% |  |  |  |
| Oxygen suppletion |  |  |  | Room air |  | Oxygen suppletion | NRB-mask/high flow O2 |
| Heart rate/min | <70 | 70-79 | 80-89 | 100-150 | 151-170 | 171-180 | >180 |
| Capillary refill time (sternal) |  |  |  | <3 seconds |  |  | ≥3 seconds |
| Blood pressure (systolic) (mmHg) | <60 | 60-69 | 70-79 | 80-100 | 101-120 | 121-150 | >150 |

*Elevated work of breathing: nasal flaring, retractions i.e. intercostal

| **Age 1 – 4 years** | | | | | | | |
| --- | --- | --- | --- | --- | --- | --- | --- |
| **Score** | **4** | **2** | **1** | **0** | **1** | **2** | **4** |
| Respiratory rate/min | <12 | 12-14 | 15-19 | 20-40 | 41-60 | 61-70 | >70 |
| Work of breathing* |  |  |  | Normal | Minimally elevated | Mildly elevated | Severely elevated and/or apnea |
| Saturation |  | <91% | 91-94% | >94% |  |  |  |
| Oxygen suppletion |  |  |  | Room air |  | Oxygen suppletion | NRB-mask/high flow O2 |
| Heart rate/min | <60 | 60-69 | 70-89 | 90-120 | 121-150 | 151-170 | >170 |
| Capillary refill time (sternal) |  |  |  | <3 seconds |  |  | ≥3 seconds |
| Blood pressure (systolic) (mmHg) | <65 | 65-74 | 75-89 | 90-110 | 111-125 | 126-160 | >160 |

*Elevated work of breathing: nasal flaring, retractions i.e. intercostal

| **Age 4 – 12 years** | | | | | | | |
| --- | --- | --- | --- | --- | --- | --- | --- |
| **Score** | **4** | **2** | **1** | **0** | **1** | **2** | **4** |
| Respiratory rate/min | <11 | 11-14 | 15-19 | 20-30 | 31-40 | 41-50 | >50 |
| Work of breathing* |  |  |  | Normal | Minimally elevated | Mildly elevated | Severely elevated and/or apnea |
| Saturation |  | <91% | 91-94% | >94% |  |  |  |
| Oxygen suppletion |  |  |  | Room air |  | Oxygen suppletion | NRB-mask/high flow O2 |
| Heart rate/min | <50 | 50-59 | 60-69 | 70-110 | 111-130 | 131-150 | >150 |
| Capillary refill time (sternal) |  |  |  | <3 seconds |  |  | ≥3 seconds |
| Blood pressure (systolic) (mmHg) | <70 | 70-79 | 80-89 | 90-120 | 121-140 | 141-170 | >170 |

*Elevated work of breathing: nasal flaring, retractions i.e. intercostal

| **Age** ≥12 years | | | | | | | |
| --- | --- | --- | --- | --- | --- | --- | --- |
| **Score** | **4** | **2** | **1** | **0** | **1** | **2** | **4** |
| Respiratory rate/min | <10 | 10 | 11 | 12-16 | 17-22 | 23-30 | >30 |
| Work of breathing* |  |  |  | Normal | Minimally elevated | Mildly elevated | Severely elevated and/or apnea |
| Saturation |  | <91% | 91-94% | >94% |  |  |  |
| Oxygen suppletion |  |  |  | Room air |  | Oxygen suppletion | NRB-mask/high flow O2 |
| Heart rate/min | <40 | 40-49 | 50-59 | 60-100 | 101-120 | 121-140 | >140 |
| Capillary refill time (sternal) |  |  |  | <3 seconds |  |  | ≥3 seconds |
| Blood pressure (systolic) (mmHg) | <75 | 75-84 | 85-99 | 100-130 | 131-150 | 151-190 | >190 |

*Elevated work of breathing: nasal flaring, retractions i.e. intercostal

**Supplemental Table 3. Current available literature on the safety of laparoscopic ovarian tissue cryopreservation in children *and* adults**

| **Paper** | **Study design** | **Sample size n (n*)** | **Median age at OTC in years* (range)** | **Indication OTC** | **Method of OTC** | **Patients with complications** | | **OTC-related mortality** | **Not OTC-related mortality including cause of death** |
| --- | --- | --- | --- | --- | --- | --- | --- | --- | --- |
|  |  |  |  |  |  | **CD grade 1-2** | **CD grade 3-4** |  |  |
| *Rosendahl et al.,* 2008 (2) | Retrospective questionnaire cohort | 92 (10) | Mean 25.4 years (9-37) | Cancer (n=86), non-malignant disease (n=6) | NA | None | Conversion^$^ (**n=1**)  WI with re-operation (**n=2**)  Bladder injury with  re-operation (**n=1**) | n=0 | NA |
| *Oktay et al.,* 2010 (3) | Prospective longitudinal cohort | 59 (19) | 26.7 (4-44) | Cancer | Unilateral complete oophorectomy | None | None | n=0 | n=30** |
| *Lawrenz et al.*, 2011 (4) | Retrospective cohort | 241 (NA) | Mean 27.4 years (15-40) | Cancer (NA), non-malignant disease (NA) | NA | Postponement of start therapy (n=1) | None | n=0 | NA |
| *Gracia et al.,* 2012 (5) | Prospective cohort | 21 (9) | NA (8-36) | Cancer (n=20), non-malignant disease (n=1) | Unilateral complete oophorectomy (n=3), ovarian biopsies (n=18) | None | None | n=0 | NA |
| *Dolmans et al.*, 2013 (6) | Retrospective longitudinal cohort | 582 (NA) | Mean 23.0 years (0.8-39) | Cancer (n=391), non-malignant disease (n=85) | NA | None | None | n=0 | NA |
| *Imbert et al.,* 2014 (7) | Retrospective cohort | 255 (NA) | NA | Cancer (n=224), non-malignant disease (n=31) | Cortical biopsies (n=NA), unilateral complete oophorectomy (n=NA) | None | Sepsis (**n=1**)^#^ | n=1^#^ | Relapse (n=28) |
| *Hourvitz et al.,* 2015 (8) | Retrospective cohort | 246 (NA) | 24.2 (2-41) | Cancer | NA | None | None | n=0 | NA |
| *Rodriguez-Wallberg et al.,* 2016 (9) | Retrospective questionnaire cohort | 1608 (477) | NA | Cancer, (NA), non-malignant disease (NA) | Unilateral oophorectomy (NA), ovarian biopsies (NA) | Mild hemorrhage (NA) | None | n=0 | NA |
| *Perelli et al.*, 2024 (10) | Retrospective cohort | 311 (NA) | NA (1.8-31) | Cancer | Bilateral biopsies (n=98), unilateral sampling (n=213) | None | Hemorrhage with  re-operation (**n=2**) | n=0 | NA |

*Unless otherwise specified **Not further specified in papers, other than that death was not related to OTC procedure ^$^Conversion from laparoscopic to laparotomic surgical technique, not further specified as to why

CD: Clavien Dindo classification grade; n: number of patients; n*: subset of sample size with age <19 years in studies with patients aged >18 years; NA: not available; OTC: ovarian tissue cryopreservation; PD: progressive disease; WI: wound infection

**Supplemental Figure 1. Patient who passed away 2 months after oophorectomy procedure – a timeline**

AKI: acute kidney insufficiency; ATG: antithymocyte globulin; BAL: bronchoalveolar lavage; Hb: hemoglobin count (x10^9^/L); MDS: myelodysplastic syndrome; NA: not available; P: platelet count (x10^9^/L); PEWS: pediatric early warning signs; WBC: white blood cell count (x10^9^/L)

**References**

1. Dors N, van Bethlehem S. Vitale functies: Dutch PEWS en SBARR zakkaart (bijlage bij Vitale functies en PEWS bij kinderen). 2023 Oct.

2. Rosendahl M, Andersen CY, Ernst E, Westergaard LG, Rasmussen PE, Loft A, et al. Ovarian function after removal of an entire ovary for cryopreservation of pieces of cortex prior to gonadotoxic treatment: a follow-up study. Hum Reprod. 2008;23:2475-83.

3. Oktay K, Oktem O. Ovarian cryopreservation and transplantation for fertility preservation for medical indications: report of an ongoing experience. Fertil Steril. 2010;93:762-8.

4. Lawrenz B, Jauckus J, Kupka MS, Strowitzki T, von Wolff M. Fertility preservation in >1,000 patients: patient's characteristics, spectrum, efficacy and risks of applied preservation techniques. Arch Gynecol Obstet. 2011;283:651-6.

5. Gracia CR, Chang J, Kondapalli L, Prewitt M, Carlson CA, Mattei P, et al. Ovarian tissue cryopreservation for fertility preservation in cancer patients: successful establishment and feasibility of a multidisciplinary collaboration. J Assist Reprod Genet. 2012;29:495-502.

6. Dolmans MM, Jadoul P, Gilliaux S, Amorim CA, Luyckx V, Squifflet J, et al. A review of 15 years of ovarian tissue bank activities. J Assist Reprod Genet. 2013;30:305-14.

7. Imbert R, Moffa F, Tsepelidis S, Simon P, Delbaere A, Devreker F, et al. Safety and usefulness of cryopreservation of ovarian tissue to preserve fertility: a 12-year retrospective analysis. Hum Reprod. 2014;29:1931-40.

8. Hourvitz A, Yerushalmi GM, Maman E, Raanani H, Elizur S, Brengauz M, et al. Combination of ovarian tissue harvesting and immature oocyte collection for fertility preservation increases preservation yield. Reprod Biomed Online. 2015;31:497-505.

9. Rodriguez-Wallberg KA, Tanbo T, Tinkanen H, Thurin-Kjellberg A, Nedstrand E, Kitlinski ML, et al. Ovarian tissue cryopreservation and transplantation among alternatives for fertility preservation in the Nordic countries - compilation of 20 years of multicenter experience. Acta Obstet Gynecol Scand. 2016;95:1015-26.

10. Perelli F, Fusi G, Lonati L, Gargano T, Maffi M, Avanzini S, et al. Laparoscopic ovarian tissue collection for fertility preservation in children with malignancies: a multicentric experience. Front Surg. 2024;11:1352698.
